# Increased FOXM1 Expression was Associated with the Prognosis and the Recruitment of Neutrophils in Endometrial Cancer

**DOI:** 10.1155/2023/5437526

**Published:** 2023-04-29

**Authors:** Jing Chen, Pusheng Yang, Shaojing Li, Yichen Feng

**Affiliations:** ^1^Department of Obstetrics and Gynecology, The Sixth People's Hospital Affiliated to Shanghai Jiaotong University School of Medicine, Shanghai, China; ^2^Department of Obstetrics and Gynecology, Shanghai Key Laboratory of Gynecology Oncology, Renji Hospital, School of Medicine, Shanghai Jiao Tong University, Shanghai, China; ^3^Department of Obstetrics and Gynecology, Shanghai Fengxian District Central Hospital, Shanghai, China; ^4^Baoshan Branch, Ren Ji Hospital, School of Medicine, Shanghai Jiao Tong University, Shanghai, China

## Abstract

**Background:**

Although the biological functions of Forkhead box protein M1 (FOXM1) were explored in a variety of cancer, to date, however, little attention has been paid to the situation of FOXM1 in EC endometrial cancer (EC).

**Method:**

Bioinformatics analysis, including GEPIA, TIMER, cBioPortal, LinkedOmics, and STRING were used to analyze the FOXM1 gene expression, genetic alteration, and immune cell infiltration in EC. IHC staining, qPCR, cell viability, and migration assay were applied to identify the functions of FOXM1 in EC.

**Results:**

FOXM1 was highly expressed in EC tissues and closely correlated with the prognosis of EC patients. FOXM1 knockdown inhibited EC cell proliferation and invasion as well as migration. FOXM1 genetic alteration was verified in EC patients. Coexpression network of FOXM1 indicated that it had roles in the EC cell cycle and the infiltration of immune cells in EC. Furthermore, bioinformatic and immunohistochemical analysis indicated that FOXM1 induced the increased CD276 expression and also enhanced the neutrophil recruitment in EC.

**Conclusion:**

Our present study discovered a novel role of FOXM1 in EC, suggesting FOXM1 could be treated as a potential prognostic biomarker and immunotherapeutic target in EC diagnosis and treatment.

## 1. Introduction

Endometrial cancer (EC) makes up 2% of all new cancer cases each year and is one of the top three diagnosed gynecological malignancies among women [1]. The incidence rate of EC has been going up worldwide, about 10%–15% of EC patients present with the advanced disease stage [2, 3]. A 5-year survival rate among EC patients with distant metastases has been reported to be 17%. Hence, it is urgent to understand the underlying pathological mechanisms during EC progression for purpose of developing more effective treatments and yielding better outcomes for EC patients.

The Forkhead box protein M1 (FOXM1), one of the Forkhead superfamily members, has an evolutionarily conserved winged-helix DNA-binding region, which is useful for its function as a transcription factor typically associated with proliferation [4]. FOXM1 plays a crucial role in cell cycle by managing the transition from G1/S to G2/M [5, 6]. It also has important functions in mitosis, because the mice with Foxm1deficiency is lethal between E14.5 and E16.5 as a result of the failure in heart and liver formation [7, 8]. As a transcription factor, FOXM1 function is managed by many different signaling pathways, such as p53, Ras, and Hedgehog, thereby activating the downstream targets [9–12], and also by different kinds of posttranslational modifications, such as phosphorylation, SUMOylation, acetylation, or ubiquitination [13].

To identify a novel promising therapeutic target or prognostic biomarker is becoming one of the hot spots for tumor therapy. More and more literature have demonstrated that FOXM1 expression is highly upregulated in multiple cancers and is also related to the decreased survival [14–17]. FOXM1 has key functions in tumor development, invasion, and metastasis, such as lung, pancreatic, as well as breast cancer [16–18]. Thus, it has been indicated that FOXM1 can be treated as a latent therapeutic target of cancer, which caused more and more attention [19, 20]. Whereas, the biological roles, as well as the underlying mechanisms of FOXM1 in EC, remain undefined yet.

In our present study, it was identified that FOXM1 was extremely highly expressed in EC and correlated with poor prognosis. We also explored the oncogenic function of FOXM1 on EC cell proliferation, migration, as well as invasion. FOXM1 had an influence on the cell cycle of the tumor cells and the immune cell infiltration in EC. Furthermore, FOXM1 enhanced CD276 expression and the neutrophil recruitment in EC. In brief, our findings unveiled a novel function of FOXM1 in EC, indicating that FOXM1 has the possibility to be treated as a prognostic biomarker as well as immunotherapeutic target in EC.

## 2. Materials and Methods

### 2.1. Data Mining

The gene expression of FOXM1 in both tumor tissues and matching normal tissues was obtained in the GEPIA2 based on TCGA and GTEx databases (http://gepia2.cancer-pku.cn/#analysis). The gene expression of FOXM1 for EC was also obtained from the Gene Expression Omnibus database (GSE17025). The association between the FOXM1 expression and the infiltrated immune cell types in EC was analyzed using the “Immune-Gene” module in the online Sanger box (http://sangerbox.com/Index) and the Tumor Immune Estimation Resource (TIMER) 2.0 database (http://timer.cistrome.org/). The genetic alterations and the interactive network of FOXM1 in EC were obtained from cBioPortal (https://www.cbioportal.org). The differentially expressed genes (DEGs) and the gene set enrichment analysis (GSEA) related to FOXM1 in EC were identified using the LinkedOmics database based on the TCGA database (http://www.linkedomics.org/login.php). A protein–protein interaction network analysis was applied to analyze the expression of FOXM1 and the potential interaction partners using STRING (https://string-db.org/).

### 2.2. Cell Culture and Regents

From American Type Culture Collection (ATCC), four human EC cell lines (ISHIKAWA, HEC-1A, ECC1, HECE-1B) were purchased. All of these cell lines were incubated at 37°C in a humidified incubator with 5% CO_2_ and cultured using the ATCC-indicated medium, which was supplemented with 1% antibiotics (100 units/ml penicillin and 100 *μ*g/ml streptomycin) as well as with fetal bovine serum (FBS, 10% v/v); negative for mycoplasma contamination of all of above cell lines were verified before using them for further assays.

### 2.3. Clinical Samples

In this study, the human EC tissue microarray, including 120 EC samples and 17 noncancerous endometrial samples, was used for FOXM1 staining [21]. To investigate the correlation between FOXM1 and CDF66b, another human EC tissue microarray, including 13 EC samples and five noncancerous endometrial samples, was purchased from Servicebio, Wuhan, China. All procedures were carried out according to the rules of the China Ethical Review Committee.

### 2.4. Construction of shRNA-FOXM1 Lentivirus Constructs

1 × 10^6^ recombinant lentivirus-transducing units containing either shRNA-FOXM1 or shRNA-control (purchased from GeneCopoeia) mixture with 5 *μ*g/ml polybrene were infected into EC cells. After 24 hr, the selected EC cells were maintained with 2 *μ*g/ml puromycin. After the section, the efficiency of FOXM1 knockdown was confirmed using qRT-PCR and WB assays. Then the cells with higher knockdown efficiency were cultured for the following experiments for the cell function.

### 2.5. Isolation of RNA and Quantitative Real-Time PCR (qPCR)

According to the manufacturer's instruction, total RNA of different EC cell lines was extracted and reversely transcribed using Trizol regent (Takara) and PrimeScript RT-PCR Kit (Takara), respectively. Applied Biosystems PRISM 7500 Sequence Detection System was used for performing real-time PCR with 2x SYBR Green qPCR Master Mix (Bimake, China) using the following settings: an initial cycle at 95°C for 1 min and 35 cycles of 15 s at 95°C ending by 30 s at 60°C. The target gene relative mRNA levels were calculated and standardized using 2^−*△△*Ct^ method relative to control gene: GAPDH. The primer sequences were used in our study as shown below:

  FOXM1-Forward, 5′-ATACGTGGATTGAGGACCACT-3′;
  FOXM1-Reverse, 5′- TCCAATGTCAAGTAGCGGTTG-3′;
  CD276-Forward, 5′- AGCACTGTGGTTCTGCCTCACA-3′;
  CD276-Reverse, 5′-CACCAGCTGTTTGGTATCTGTCAG-3′;
  GAPDH-Forward, 5′- GCACCGTCAAGGCTGAGAAC -3′;
  GAPDH-Reverse, 5′- ATGGTGGTGAAGACGCCAGT -3′.

### 2.6. Western Blotting (WB)

RIPA buffer with 1 mM PMSF and protease inhibitor cocktail was used to lyse EC cells. Then proteins contained in EC cell lysis solution were divided using accurate SDS-PAGE gel. After transferring onto nitrocellulose membrane (Shaanxi, China), the protein was blocked by 1% BSA within PBS/Tween at room temperature (RT) for 1 hr. Next, the membrane was further cultured overnight at 4°C with anti-FOXM1 (1 : 1,000, ab207298) or anti-GAPDH (1 : 1,000 dilution, Santa Cruz). After washing, the membranes were further incubated using corresponding secondary antibodies, and the Odyssey imaging system (LI-COR Biosciences, Lincoln, NE) was used for detecting targeted protein signals.

### 2.7. Viability of Cell and Formation of Colony Assay

For the viability of cell assay, 1,500 EC cells per well were added into a 96-well plate and incubated using 100 *μ*l complete culture medium for 24, 48, 72, 96, and 120 hr. Each well medium was replaced at the indicated time points with solution of 10% (v/v) cell counting kit-8 (CCK-8; Share-Bio, China). After 1 hr of incubation, colorimetric dye that was produced by metabolized CCK-8 was scaled at 450 nm by a Power Wave XS microplate reader (BIO-TEK). For the colony formation assay, 1,000 EC cells per well were cultured within a six-well plate for 14 days in the cell culture medium. After staining with 0.1% crystal violet and counting, cell colonies were statistically analyzed.

### 2.8. Migration and Invasion of Cell Assay

For the cell migration assay, 2 × 10^4^ cells contained in 200 *μ*l medium serum-free medium were put into the upper chambers of a 24-cell plate with 8 *μ*m pore size cell culture inserts (Millipore, USA). After migrating for 24 hr at 37°C, 4% paraformaldehyde (PFA) and 0.1% crystal violet were used to fix and stain the migrated cells respectively. The migrated cells in six random fields under a light microscope were counted and statistically analyzed.

For the cell invasion assay, the Matrigel was put into the upper chamber of the inserts mixed with the serum-free medium, and then 5 × 10^4^ EC cells were put onto the Matrigel and migrated for 48 hr. After invading through the membrane and fixing with 4% PFA, the cells were stained using 0.1% crystal violet and analyzed.

### 2.9. Immunohistochemistry (IHC) Staining

As described previously in literature, immunohistochemistry (IHC) staining was performed [22]. In brief, the paraffin-embedded EC and normal tissue slides were deparaffinized and rehydrated, and then blocked using 10% BSA in PBS for 30 min. Next, the sections were incubated using different primary antibodies (FOXM1, Abcam ab207298; CD66b, Biolegend 305102) overnight at 4°C, followed by incubation using the matching secondary antibodies for 1 hr at RT. Then DAB substrate kit (Thermo Scientific) was used for treating the sections and hematoxylin staining for nuclear counterstaining. Lastly, a microscope (Carl Zeiss) was used for photography and all the sections were analyzed.

### 2.10. Statistical Method

Statistical analyses were performed using GraphPad Prism 9 (San Diego, CA) in this study. Means ± standard deviation (SD) was the presented form of data. Comparisons between the two groups were carried out by Student's *t*-test. *P* < 0.05 was considered statistically significant.

## 3. Results

### 3.1. FOXM1 Expression Is Highly Increased in EC and Closely Associated with Poor Prognosis of EC Patients

Based on the CCLE database, we found that mRNA of FOXM1 was diffusely detected in diverse tissues (Figure S1a). next, the mRNA expression level of FOXM1 in pan-cancer through TCGA and GTEx database was analyzed for checking the expression of FOXM1 in different types of cancer. The result displayed that FOXM1 mRNA was clearly upregulated in most kinds of cancers, indicating the vital functions of FOXM1 on the occurrence and progression of tumors (Figure S1b). Furthermore, we explored that FOXM1 high expression level predicted bleak prognosis in most types of cancer (Figure S1c).

**Figure 1:**
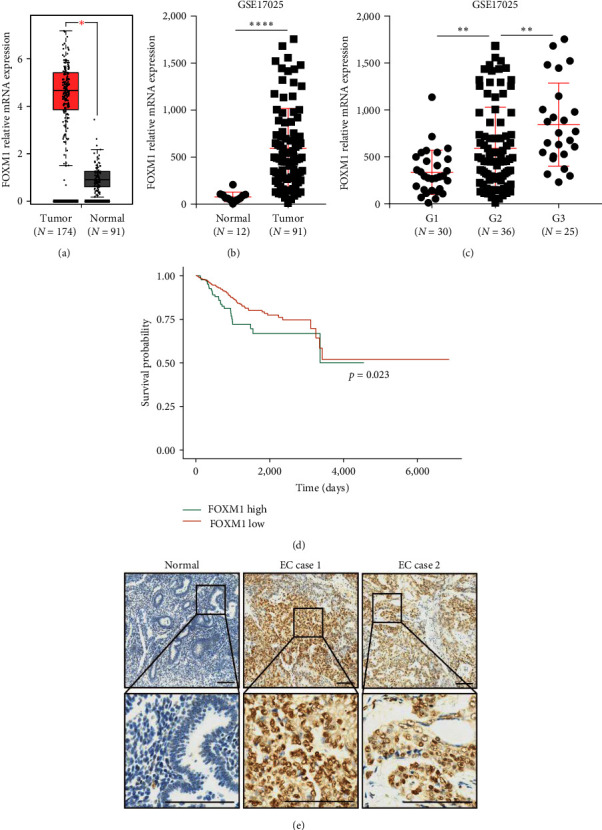
Expression of FOXM1 expression is significantly increased and closely correlated with bleak clinical outcome in endometrial cancer (EC) patients. (a) Difference of FOXM1 mRNA expression was analyzed between EC tissues and health tissue in TCGA database. (b and c) Difference of FOXM1 mRNA expression in EC patients was analyzed using GEO database GSE17025. (d) Overall survival ratio was detected by Kaplan–Meier analysis according to expression of FOXM1 in EC patients using TCGA database. Log-rank test was using for calculating *p*-value. (e) Representative IHC staining showing the FOXM1 expression in normal endometrium samples and two EC samples. Scale bar = 100 *μ*m.  ^*∗*^*p* < 0.05,  ^*∗∗*^*p* < 0.01,  ^*∗∗∗∗*^*p* < 0.0001.

In EC, the mRNA expressed level of FOXM1 was highly increased in EC tissues in comparison with corresponding health tissues (Figure 1(a)). Additionally, the gene expression data from GEO database GSE17025 displayed that FOXM1 mRNA expression level was significantly increased in EC and also was positively associated with EC pathological grades (Figures 1(b) and 1(c)). Highly expressed FOXM1 was indicated by Kaplan–Meier analysis significantly correlated with bleak prognosis in EC patients (Figure 1(d)). Furthermore, immunohistochemical staining of EC tissues indicated that the protein level of FOXM1 was clearly increased in EC tissues in comparison with the matching health tissues (Figure 1(e)).

### 3.2. Proliferation, Migration, as well as Invasion of EC Cells Was Promoted by FOXM1 In Vitro

**Figure 2:**
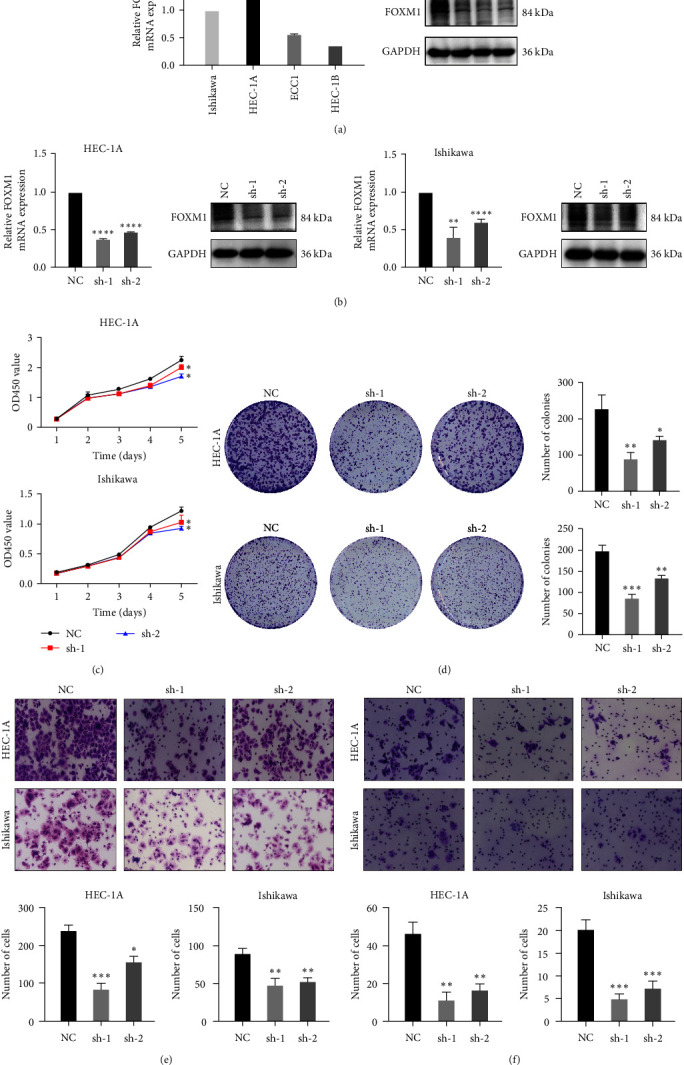
Proliferation, migration, as well as invasion of EC cells were promoted by FOXM1. (a) The difference of FOXM1 expression on mRNA as well as protein level were detected by qPCR and WB assay between four different EC cell lines. (b) The knockdown efficacy of FOXM1 by shRNA in two EC cell lines were confirmed in both mRNA and protein level by qPCR and WB. (c and d) The cell growth analysis in two EC cell lines with or without FOXM1 knockdown were analyzed by CCK8 and clone formation assay. (e and f) The migration as well as invasion of two EC cell lines with or without FOXM1 knockdown were analyzed. The quantification was performed from six randomly selected fields. Means ± standard deviation (SD) was used for presenting analyzed data.  ^*∗*^*p* < 0.05,  ^*∗∗*^*p* < 0.01,  ^*∗∗∗*^*p* < 0.001,  ^*∗∗∗∗*^*p* < 0.0001.

To evaluate the role of FOXM1 within EC development, the mRNA and protein expressed levels of FOXM1 were first tested in four cell lines of EC including ISHIKAWA, HEC-1A, ECC1, and HECE-1B by qPCR and WB. Among them, ISHIKAWA and HEC-1A cells showed relatively higher mRNA and protein expression of FOXM1, which were chosen for the subsequent experiments (Figure 2(a)). In order to verify the oncogenic functions of FOXM1 in EC, we established stable EC cell lines with knockdown of FOXM1 by short hairpin RNA (shRNA) in ISHIKAWA as well as HEC-1A cells, which were verified by qPCR and WB analyses (Figure 2(b)). The FOXM1 knockdown significantly inhibited the proliferation of the two EC cell lines above as shown by CCK8 as well colony formation assay (Figures 2(c) and 2(d)). What's more, the migration as well as invasion were also suppressed by FOXM1 knockdown in both ISHIKAWA and HEC-1A cells (Figures 2(e) and 2(f)).

### 3.3. The Genetic Alteration and the Interaction Networks of FOXM1 in EC Patients

**Figure 3:**
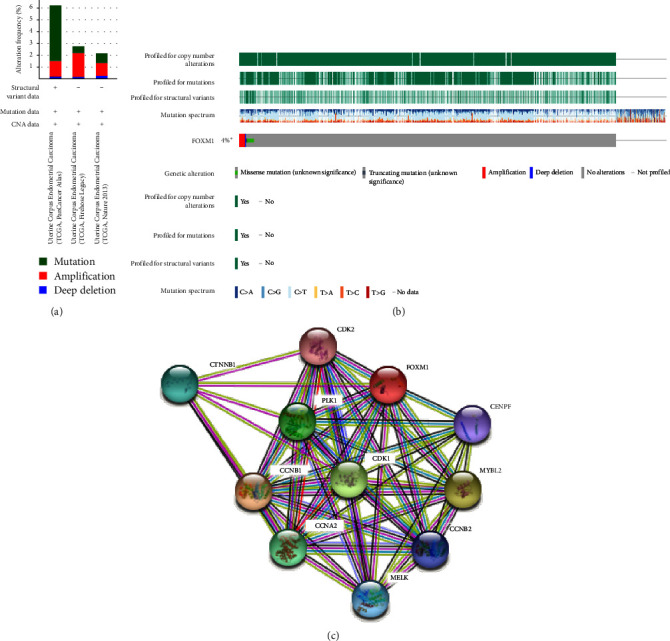
FOXM1 genetic alteration and analysis in EC patients. (a and b) The FOXM1 genetic alteration in EC patients using TCGA database. (c) STRING approach showed protein–protein network of FOXM1 in EC patients.

The genetic alterations, correlations, and interaction networks of FOXM1 were analyzed in EC based on the TCGA database using the cBioPortal online tool. Two or more alterations were detected in EC using the TCGA database (Figure 3(a)). FOXM1 was altered in 4% of patients with EC (Figure 3(b)). In addition, the protein–protein interaction networks were analyzed for exploring possibility of proteins with FOXM1 using the STRING approach. The interaction network showed that FOXM1 was closely correlated with those proteins who are involved in the cell cycle, such as cyclin-dependent kinase 1 (CDK1), CDK2, Cyclin B1 (CCNB1), as well as CCNB2, indicating FOXM1 may act as an important regulator of the tumor cell cycle (Figure 3(c)). Those might be closely associated with FOXM1-related molecular functions.

### 3.4. The Coexpression Analysis of FOXM1 in EC

**Figure 4:**
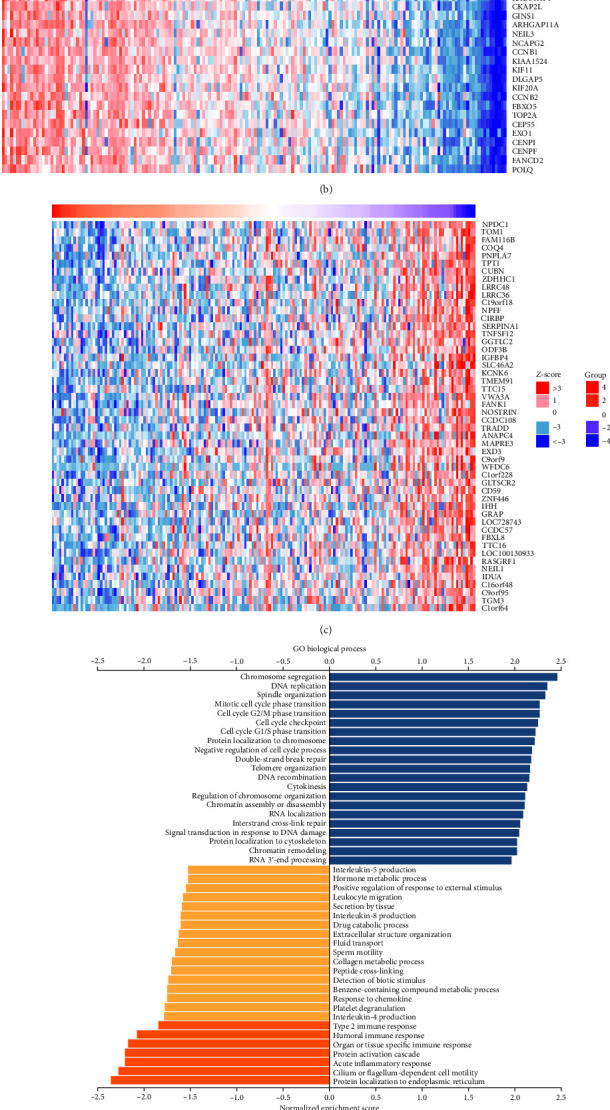
FOXM1 coexpression genes in EC analyzed by online LinkedOmics approach from TCGA database. (a) All FOXM1 significantly correlated genes in EC as shown in volcano plot. Positively correlated genes displayed as red dots, negatively correlated genes displayed as green dots, and no correlated genes with FOXM1 displayed as black dots. (b and c) Top 50 positive or negative relative genes of FOXM1 in EC are shown in the heatmap. (d and e) FOXM1 and its coexpression genes in EC analyzed by GO biological process and KEGG pathway approaches.

In order to gain deeper understanding of the exact function as well as the potential mechanism of FOXM1 in EC, the coexpression network of FOXM1was identified in EC using the LinkedOmics database (LinkFinder module) and found that 4,001 genes (red color dots) were positively associated with FOXM1, and 3,791 genes (green color dots) were negatively associated with FOXM1 (Figure 4(a)). The top positive and negative 50 genes associated with FOXM1 were shown in the heatmaps, respectively (Figures 4(b) and 4(c)). These highly coexpressed genes of FOXM1, studied by GO biological and KEGG pathways analysis, were largely gathered to different progress linking to cell cycle, including DNA replication, cell cycle G2/M phase transition, cell cycle checkpoint, and mitotic cell cycle phase transition (Figures 4(d) and 4(e)). Besides, coexpressed genes of FOXM1 also participated in leukocyte migration, response to chemokines, cell adhesion molecules, acute inflammatory response, etc. (Figures 4(d) and 4(e)). Taken together, those analyses indicated that FOXM1 has influence on EC cell cycle and the infiltration of immune cells in EC.

### 3.5. FOXM1 Enhances the CD276 Expression in the Tumor Cells and the Neutrophil Infiltration in the Tumor Microenvironment of EC

**Figure 5:**
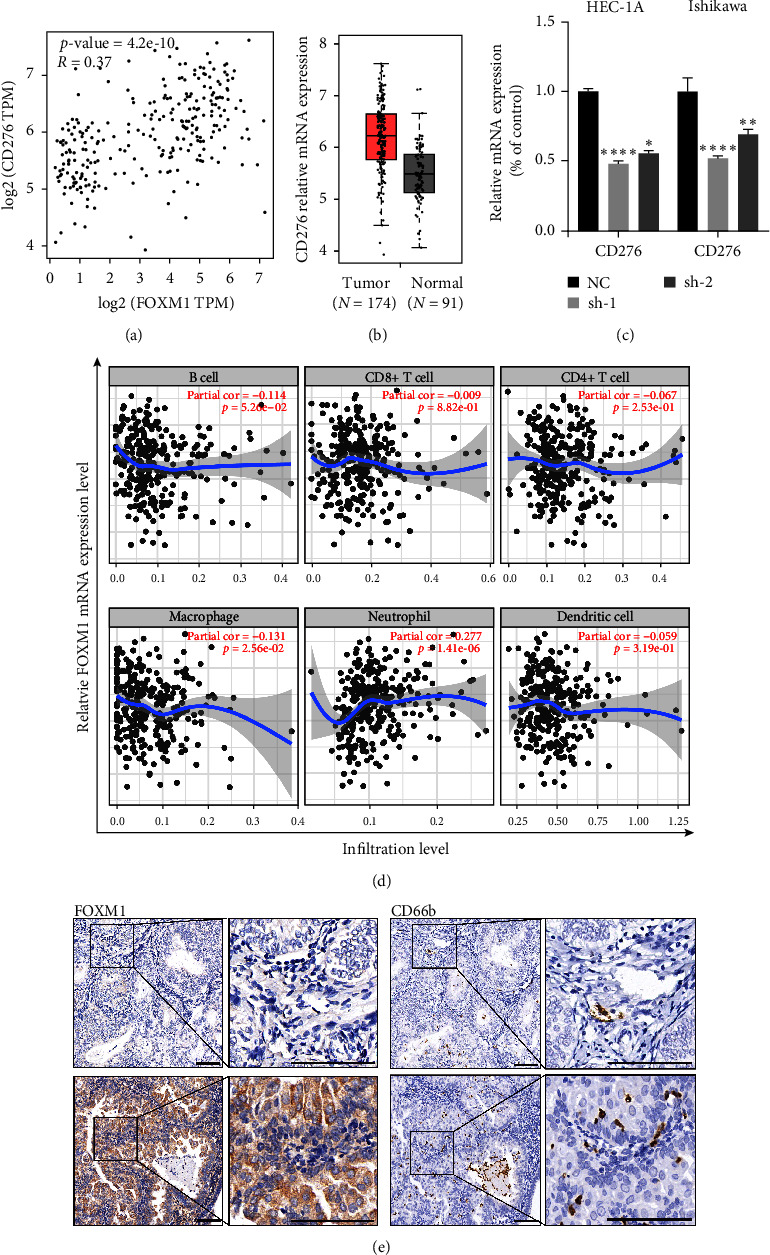
FOXM1 enhanced CD276 expression in EC cells and was closely associated with the infiltration of neutrophils in EC samples. (a) The correlation analysis of FOXM1 with CD276 expression in EC using TCGA dataset. (b) Difference of CD276 mRNA expression level between EC tissue and normal tissue was verified using TCGA database. (c) FOXM1 and CD276 relative mRNA expression situations in two cell lines of EC with or without FOXM1 knockdown.  ^*∗*^*p* < 0.05,  ^*∗∗*^*p* < 0.01,  ^*∗∗∗∗*^*p* < 0.0001. (d) Analysis of correlation between the FOXM1 mRNA level and infiltrated immune cells in EC using TIMER2.0. (e) Representative FOXM1 and CD66b (neutrophil marker) IHC staining in EC tissue samples with low- (upper) or high- (down) expressed FOXM1 levels. Scale bar = 100 *μ*m.

To check whether FOXM1 was involved in the immune microenvironment of tumor or not, the “Immune-Gene” analysis was performed using the Sanger box and GEPIA2 database, which showed that FOXM1 has a strong positive correlation with CD276 expression (Figure 5(a), Figure S2a). Also, mRNA of CD276 was notably upregulated in EC tissues in comparison with corresponding health tissues (Figure 5(b)). Furthermore, FOXM1 knockdown remarkedly suppressed CD276 expression not only in ISHIKAWA but also in HEC-1A cell lines (Figure 5(c)). Next, we further explored a significantly strong association between FOXM1 and neutrophils (Figure 5(d), Figure S2b). Therefore, we checked the neutrophil infiltration in EC tissue microarray. IHC assay displayed that a number of infiltrated neutrophils in the tumor microenvironment were positively associated with the expression of FOXM1 in EC (Figure 5(e)). Taken together, FOXM1 may contribute to regulating the tumor immune microenvironment through neutrophil infiltration in EC.

## 4. Discussion

FOXM1, as an oncogenic transcription factor, plays vital functions in regulating various biological processes, the dysregulation and activation of FOXM1 cause tumor progression; it is also involved in epithelial–mesenchymal transition (EMT), metastasis, invasion, as well as carcinogenesis [23, 24]. As indicated by a recent meta-analysis, FOXM1 can be used as a significant prognosticator for the bleak clinical outcome in many cancers [25]. Our study aims to explore the molecular mechanism for FOXM1 in EC and provide the potential diagnostic and therapeutic biomarker or target for EC.

Our previous study has shown that FOXM1 affected the metabolic activity of EC cells via the FOXM1-SLC27A2 signaling pathway to promote EC [21]. Also, FOXM1 plays crucial functions in aerobic glycolysis in pancreatic cancer via transcriptional regulating LDHA function [26]. However, in this study, we uncovered a new function of FOXM1 in EC progress. It is well known that FOXM1 plays a key function in regulation of the G1 and S phase transition or between G2 and M phase transition [27, 28]. By using the cBioPortal online tool, we found the potential proteins that interacted with FOXM1, which were cell cycle-related proteins, such as CDK1, CDK2, CCNB1, and CCNB2, indicating that FOXM1 might play its role through interacting with those proteins. This point requires further investigation. Coexpression network analysis of FOXM1 by GO biological and KEGG pathways analysis suggested that FOXM1 was not only involved in the cell cycle but also had impact on the recruitment of different types of immune cells in EC.

Tumor microenvironment (TME) is important in the majority of cancer cell functions such as proliferation, migration, as well as metastasis, which has an important role in the progression and recurrence of tumors. TME consists of different kinds of components, including tumor cells, endothelial cells, fibroblasts, many types of immune cells, and further including cytokines, growth factors, as well as extracellular matrix [29–31]. More and more evidence showed that TME immune cells play crucial functions in either promoting or suppressing tumor progression [32–34]. The present study indicated that FOXM1 has a significantly strong association with neutrophils in EC.

Neutrophils, which belong to the innate immune cells and are also the most abundant leukocytes in the circulation, are the first barrier of defense in opposition to bacterial and fungal infections and also play crucial roles to respond to various inflammatory factors, leading to tumor progression and metastasis [35, 36]. By IHC staining, we verified that the expression level of FOXM1 in EC was positively related to the infiltration of neutrophils in EC. It will be better to investigate this using animal models *in vivo*. We can perform subcutaneous tumor formation in nude mice by human EC cell line. However, it is not possible to check the neutrophils using the above model as the nude mice are lacking the immune system. In addition, due to the lacking of mouse EC cell lines, it is also not possible to investigate the FOXM1 function in neutrophil recruitment in mouse models. Therefore, how FOXM1 affects the neutrophil recruitment in the TME of EC and what is the underlying mechanism are still not known and need to be further investigated.

One of the most critical elements guarding against the occurrence and progression of tumors is immunosurveillance [37]. CD276 is an important immune checkpoint molecular, which is highly expressed in many solid tumors and is relatively low in most normal tissues. Furthermore, CD276 was reported negatively related to bleak prognosis as well as clinical outcomes of tumor patients with many kinds of cancer [38–41]. Here, the expression level of CD276 was verified clearly to be upregulated in EC patients. By “Immune-Gene” analysis and the following experimental verification, we demonstrated that FOXM1 expression was closely correlated with CD276 expression level. More studies focus on interaction between FOXM1 and CD276 in EC should be investigated with more details in the future.

## 5. Conclusion

In conclusion, we indicated that the FOXM1 expression level was significantly increased in EC and closely associated with bleak prognosis of EC patients. FOXM1 knockdown inhibited the proliferation, migration, as well as invasion of EC cells. Coexpression network analysis showed that FOXM1 participated in the tumor cell cycle and the infiltration of immune cells in EC. We further found that FOXM1 induced the increased expression of CD276 and also enhanced the neutrophil recruitment in EC. Taken together, our findings in this study indicate that FOXM1 can be treated as a promising diagnostic biomarker as well as immunotherapy target in EC patients.

## Data Availability

All the data supporting the findings of this study are available within the article and its supplementary information files or can be obtained from the corresponding authors upon reasonable request.

## Supplementary Materials

Supplementary Materials Figure S1: The analysis of FOXM1 mRNA expression and its correlation with survival situation in many types of cancer. Figure S2: Analysis of tumor immune microenvironment correlated with FOXM1 in EC using the Sanger box. Figures S1 and S2 show the expression analysis of FOXM1 and the tumor immune microenvironment analysis related to FOXM1, respectively.
