# Supplementary material for: Increased FOXM1 Expression was Associated with the Prognosis and the Recruitment of Neutrophils in Endometrial Cancer
Source: J Immunol Res. 2023 Apr 29;2023:5437526. doi: 10.1155/2023/5437526 (PMC10163965; doi:10.1155/2023/5437526)
Supplement: Supplementary Materials — Figure S1: The analysis of FOXM1 mRNA expression and its correlation with survival situation in many types of cancer. Figure S2: Analysis of tumor immune microenvironment correlated with FOXM1 in EC using the Sanger box. Figures S1 and S2 show the expression analysis of FOXM1 and the tumor immune microenvironment analysis related to FOXM1, respectively. [file 5437526.f1.docx]

**Supplementary Materials**


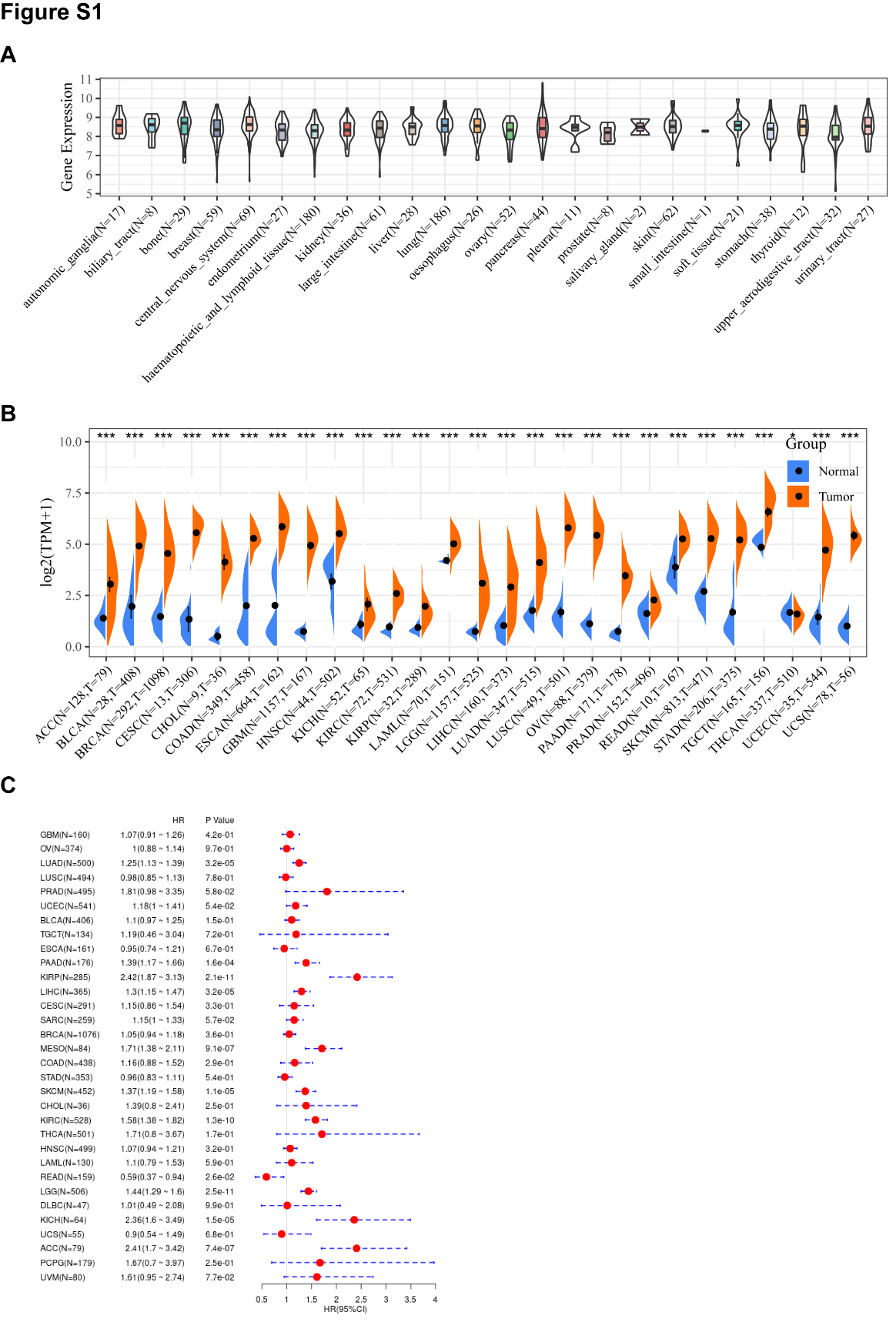


**Figure S1**. The analysis of FOXM1 mRNA expression and its correlation with survival situation in many types of cancer. (A) The mRNA of FOXM1was analyzed in diverse tissues. (B) The FOXM1 mRNA expression difference between tumor tissues and health tissues in various of cancer. (C) Association of between expression level of FOXM1 and situation of survival in various of cancer.


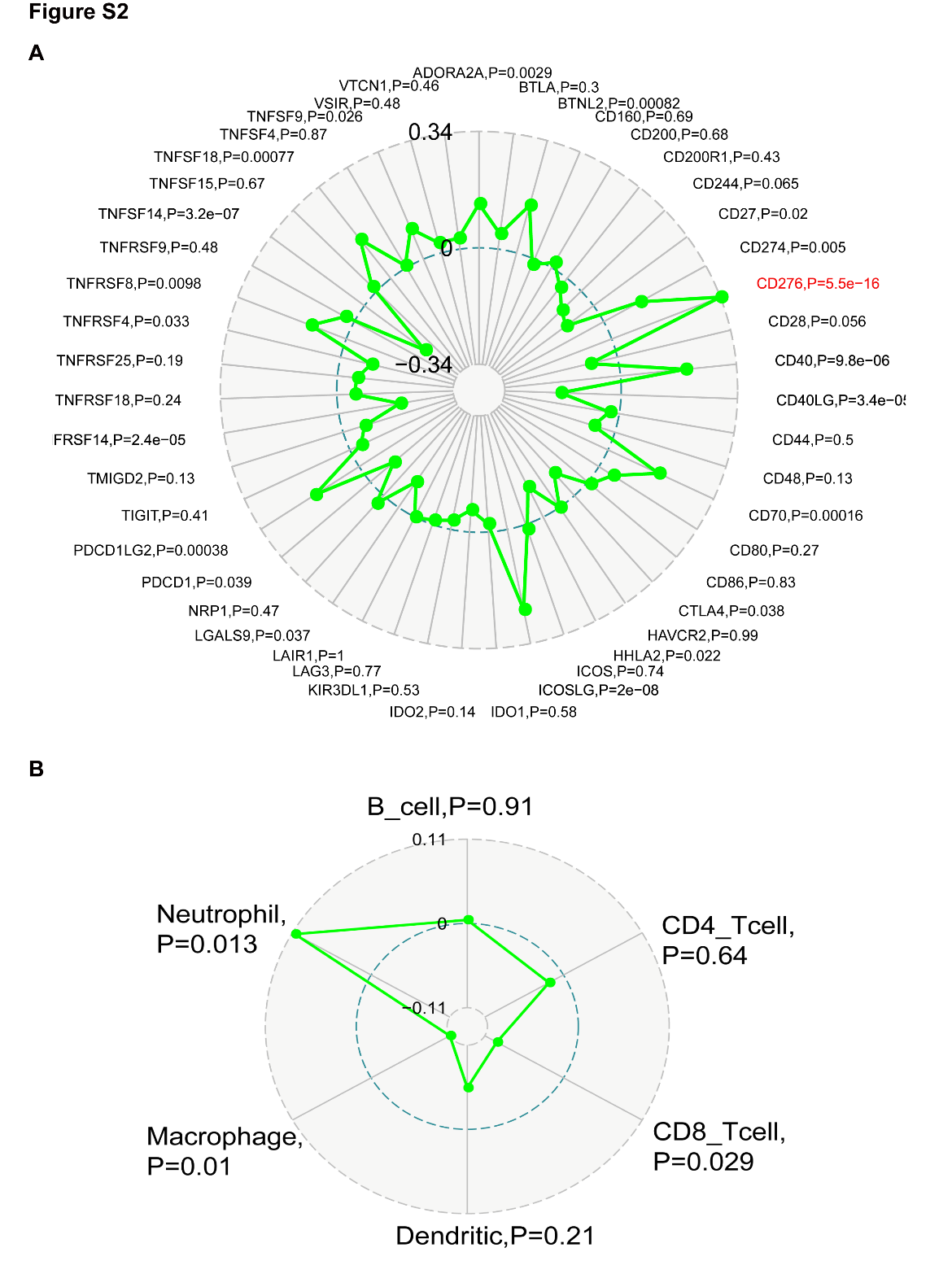
**Figure S2.** Analysis of tumor immune microenvironment correlated with FOXM1 in EC using the Sanger box. (A) Gene-immune of FOXM1 in EC was analyzed. (B) Association of FOXM1 expression with infiltrated immune cells.
